# A new genus, Nothovernonia, from tropical Africa (Asteraceae or Compositae, Vernonieae)

**DOI:** 10.3897/phytokeys.3.1131

**Published:** 2011-05-30

**Authors:** Harold Robinson, Vicki A. Funk

**Affiliations:** Department of Botany, MRC 166, US National Herbarium, National Museum of Natural History, P.O. Box 37012, Smithsonian Institution, Washington, DC. 20013–7012

**Keywords:** Compositae, *Nothovernonia*, new genus, tropical Africa, Centrapalinae, Erlangineae, phylogeny

## Abstract

*Nothovernonia* **gen. nov.**, is described as a new genus for the tropical African *Vernonia purpurea* Sch.Bip. ex Walp. and *Vernonia amblyolepis* Bak, having cymiform inflorescences, pedunculate heads with differentiated foliiform bracts at the base, apiculate involucral bracts with scarious lateral margins, spiculiferous corolla lobes, and strongly spinose, sublopohate tricolporate pollen with the colpi meeting at the poles. The new genus belongs to the subtribe Centrapalinae and a key to the known DNA-sequenced genera of the subtribe is provided. The new species names are *Nothovernonia purpurea* (Sch.Bip. ex Walp.) H.Rob. and V.A.Funk, **comb. nov.**, and *Nothovernonia amblyolepis* (Baker) H.Rob. & V.A.Funk, **comb. nov.**

## Introduction

Many genera have been named in the tribe Vernonieae over the last 200 years, see (Jones (1979b, 1981) and Jeffrey (1988) for citations of many of the names. During that time, some genera have been accepted when based on distinctions involving zygomorphic corollas (*Elephantopus* L, *Pseudelephantopus* Rohr or *Stokesia* L’Hér.), paleaceous receptacles (*Lepidonia* S.F. Blake, *Centauropsis* Boj. in DC.), syncephaly (*Elephantopus Eremanthus* Less., *Lychnophora* Mart.), a modified or reduced pappus (*Elephantopus*, *Pseudelephantopus*, *Sparganophorus* Vaill. ex Crantz, *Pacourina* Aubl., *Centratherum* Cass., *Harleya* S.F. Blake), or even sclerified tails on the anther when combined with strongly scandent habit and highly deciduous involucral bracts (*Piptocarpha* R. Br.). However, many other new genera whose members have some more general characters such as a capillary pappus, discrete heads, actinomorphic corollas, including many woody or arborecent types have not been accepted: *Gymnanthemum* Cass., *Critoniopsis* Sch. Bip., *Strobocalyx* (Blume ex DC.) Spach. Even some genera that were based on lophate pollen (*Ambassa* Steetz in Peters, *Crystallopollen* Steetz in Peters = *Polydora* Fenzl) have been summarily reduced to synonymy under the large, geographically improbable, core genus *Vernonia* Schreb. As a result, *Vernonia* s.l. was traditionally defined by what it “was not” rather than by what it “was” (Jones 1977, 1979b, 1981, Jeffrey 1988). Detailed morphological work and molecular phylogenics have finally forced the dismemberment of that unnatural genus, and true *Vernonia* s.s. is now known to be part of native floras only in the Americas (Robinson 1999a, b). As a result, the many paleotropical species that have been placed in the genus *Vernonia* are gradually being shifted into other genera (Robinson 1999a, 2007, Keeley and Robinson 2009). Although much progress has been made, there are still unplaced species, some of which can be assigned to new genera with reasonable assurance even though the defining characters are not necessarily macroscopic. One such genus is described here for a pair of species in tropical Africa including the common *Vernonia purpurea* Sch.Bip. ex Walp.

**Figure 1.**
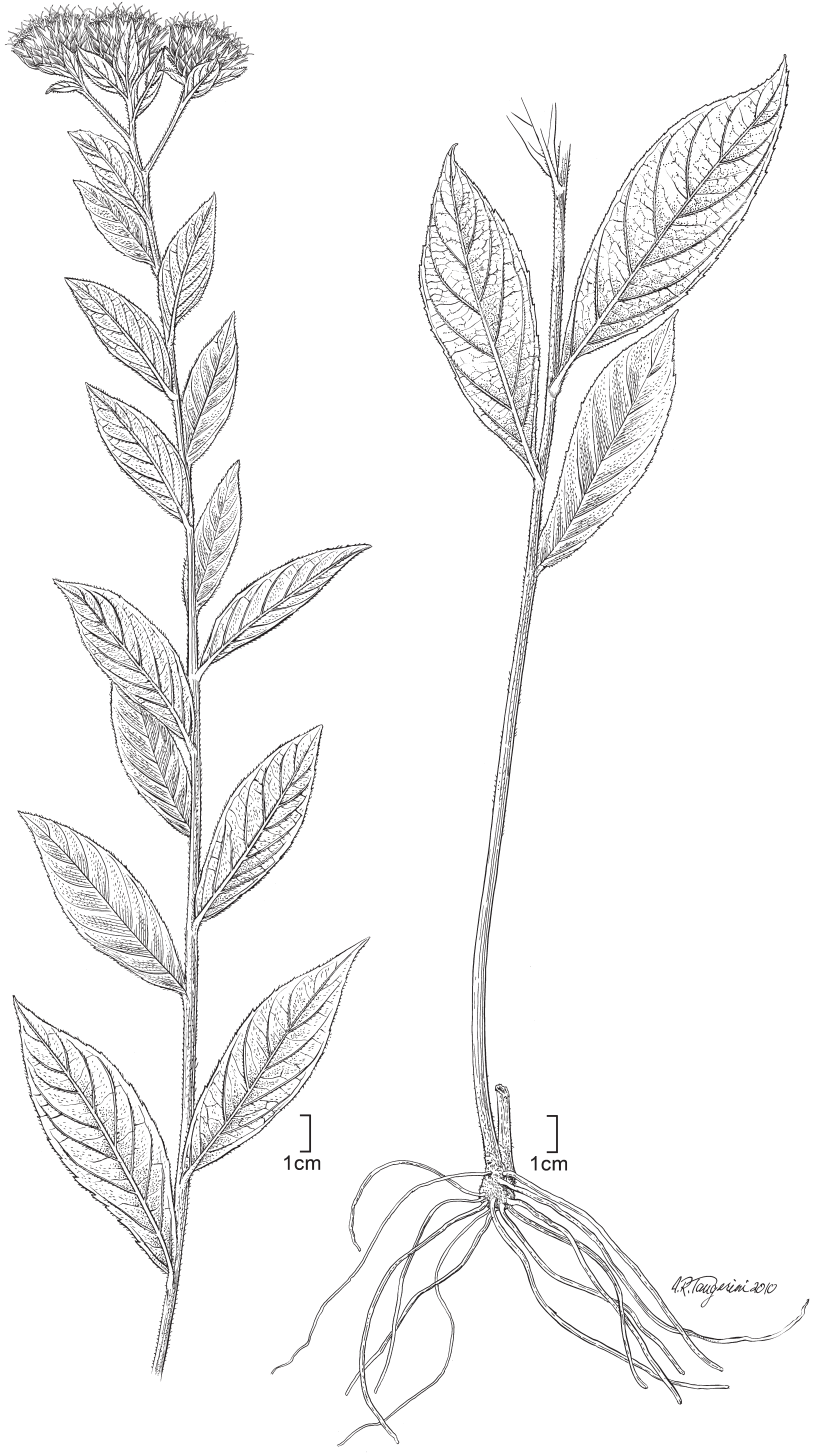
*Nothovernonia purpurea* (Sch.Bip. ex Walp.) H. Rob. and V.A. Funk: habit. [Illustration by Alice Tangerini (US)]

The species known as *Vernonia purpurea* is a coarse herb (Fig. 1) placed by Jeffrey (1988) in his *Vernonia* Subgroup A, a subgroup that included the genera *Centrapalus* Cass. and *Vernonella* Sond. as synonyms. More recent studies have shown that both *Centrapalus* and *Vernonella* are valid genera that apparently belong to different subtribes, *Centrapalus* to the Centrapalinae and *Vernonella* to the Linziinae (Robinson and Skvarla 2010). The same study has shown that *Vernonia purpurea* does not belong to either of the latter genera, differing in corolla color, corolla lobe pubescence, sweeping hairs of the style, and pollen structure. Isawumi (2008) placed *Vernonia purpurea* in the genus *Linzia* Sch.Bip. ex Walp., but *Vernonia purpurea* has neither the denticulations on the lateral margins of the involucral bracts, the bluish corollas, nor the lophate pollen with mural spurs projecting into the colpi that are characteristic of *Linzia*.

The goal of this study is to recognize the new genus and provide the proper nomenclature for a larger study on the subfamily Cichoroideae.

## Methods

The morphology was studied using herbarium material, most of which was from the U.S. National Herbarium in Washington, D.C. Microscopic characters were examined via plant material mounted on microscope slides in semi-permanent, water misable Hoyer’s Solution (Anderson 1954). Pollen grains were mostly treated with acetolysis (Erdtman 1960), followed by staining with Osmium-thio-carbohydrazide solutions and sputter coating with gold/palladium (Robinson and Skvarla 2006, 2007, Robinson et al. 2008). Unacetylized grains were rehydrated in water or alcohol directly from herbarium sheets and similarly sputter coated. Observations were made with a JEOL 880 (Samuel Roberts Microscopy Laboratory, University of Oklahoma), or a LEICA 440 and AMRAY 1810 (National Museum of Natural History, Washington DC) scanning electron microscopes (SEM), all equipped with lanthanum hexaborate (LaB6) electron sources.

Scanned images of the syntypes of *Vernonia amblyolepis* Baker were sent by the Herbarium at the Royal Botanic Gardens, Kew.

**Figure 2.**
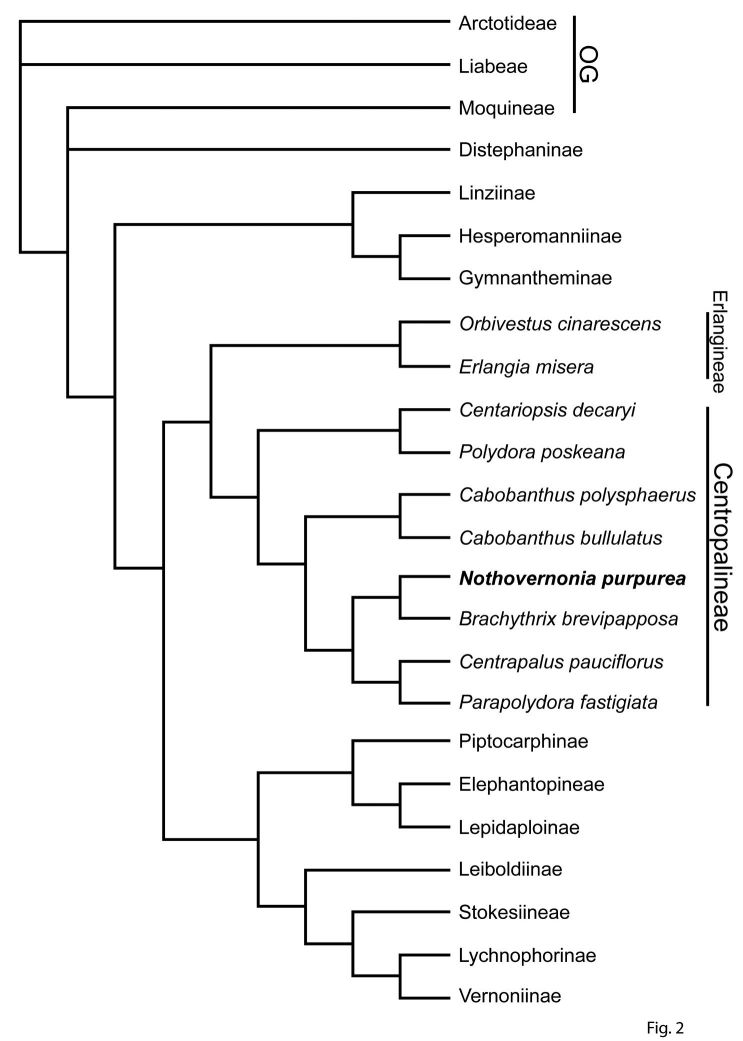
A phylogeny of the subtribes of the Vernonieae, with a detailed look at the subtribe Centropalineae. This tree is the strict consensus tree from a PAUP analysis that produced 28 trees. The data included were ITS, *ndhF, trnL-F*, and *matK*. The analysis included all known sequenced genera of the Centropalineae and is part of a larger analysis of the subfamily Cichorioideae.

The position on the molecular phylogeny was determined by analyzing DNA sequence data of ITS, *ndhF*, *trnL-F* and *matK*. The details of the molecular work will be published as part of a larger study of the subfamily Cichorioideae (Funk and Chan 2009, unpublished data, Funk pers. comm.) (Fig. 2). Before the subfamily paper can be submitted, some nomenclatural issues must be straightened out, hence the necessity of this paper.

## Discussion

**Figure 3.**
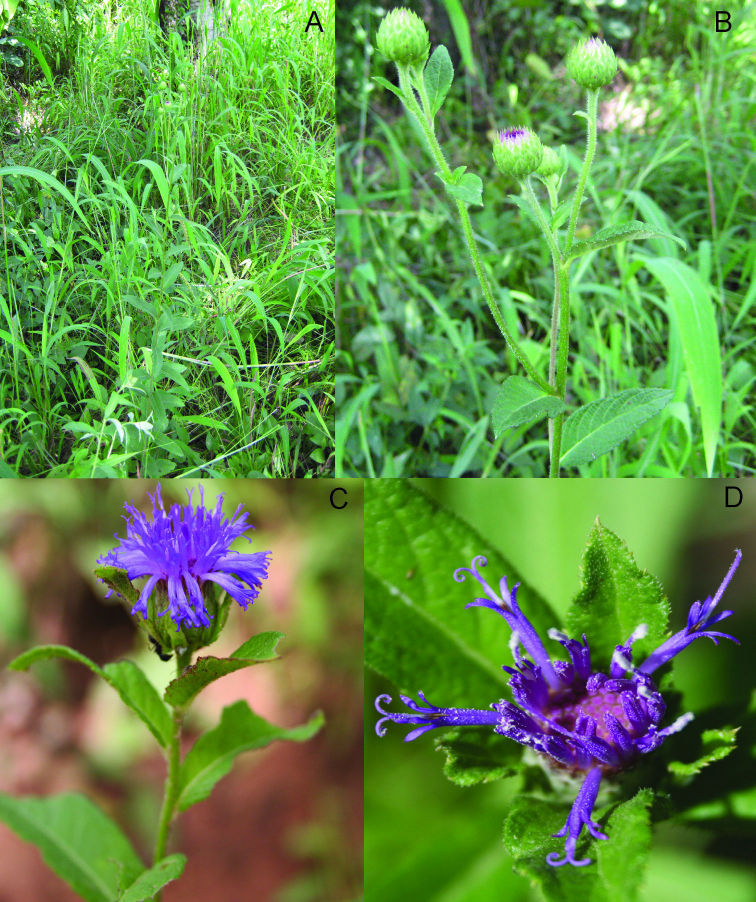
Images of *Nothovernonia purpurea* (Sch.Bip. ex Walp.) H. Rob. and V.A. Funk **A** Habitat **B** Head before flowering with outer bracts tightly appressed **C** Fully flowering head showing well developed bracts **D** Head with only a few flowers but showing the well developed bracts. [Photographs by A. Thiombiano, M. Schmidt, and K. Schumann]

The new genus, named here as *Nothovernonia*, can be distinguished from *Centrapalus* by the erect rather than decumbent bases of the stems, the apiculate laterally scarious rather than long herbaceous non-scarious tips on the involucral bracts, and the purple or lilac rather than blue-purple corollas (Fig. 3, B-D). The corolla color may be most useful in the field, with *Centrapalus* usually being bluish and *Nothvernonia* apparently never being blue. The stout spreading sweeping hairs of the style branches are different from the slender usually more appressed hairs in *Centrapalus*. The achenes are similar in having narrow though small raphids, many idioblasts on the surface, setulae with pairs of cells usually separated to a third or more of their length, but differ in the cells of the setulae being fused in the basal one to two thirds and the pappus being white to rufous. In *Centrapalus* the cells of the setulae are separate and essentially solitary to near the base, and the pappus is sordid grayish.

**Figure 4.**
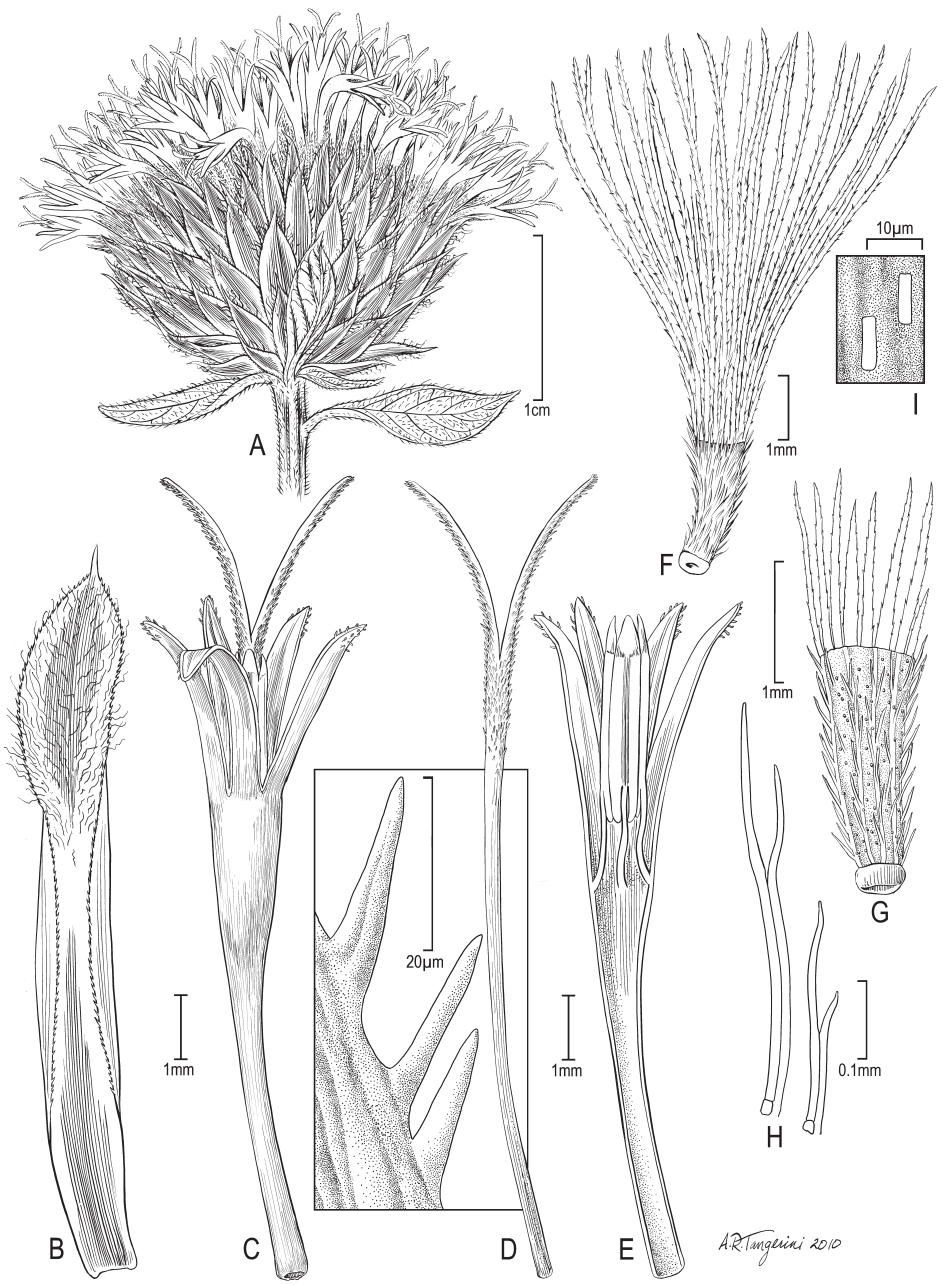
*Nothovernonia purpurea* (Sch.Bip. ex Walp.) H. Rob. and V.A. Funk **A** head **B** involucral bract **C** floret with style branches **D** style branch with detail of sweeping hairs in square **E** longitudinal section of floret showing anthers **F** achene with pappus **G** achene **H** detail of achene setulae **I** examples of raphids from achene wall. [Illustration by Alice Tangerini (US)]

Robinson and Skvarla published images of the pollen of *Nothovernonia* (Robinson and Skvarla 2010, Fig 5 A-C) and *Centrapalus* Cass. (Robinson and Skvarla 2010, Fig 3 A-D); at this time there are no images available for *Parapolydora* H. Rob. or *Brachythrix* Wild & G.V. Pope. The two species treated here in *Nothovernonia* have pollen that is similar to but not identical with *Centrapalus*. They have long spines arranged in a sublophate pattern that is much less irregular than *Cenrapalus* and the colpi are not truncated, rather they reach to and meet at the poles (Robinson and Skvarla 2010) This type of pollen is widely distributed in the tribe and of uncertain use at this point. Never-the-less, pollen is frequently very useful in the tribe and it is essential to document the characters of the pollen for future comparative studies.

The two species treated here have their corolla color and stem bases more like those of *Vernonella*, but the colpi of the pollen are not truncated and thus failing to reach the poles, the involucral bracts are not scarious across the tips, the corolla lobes are spiculiferous with stiff hairs outside distally, and the achenes have setulae with cell pairs separated nearly half way but not to the base. In addition, the two species differ from both *Centrapalus* and *Vernonella* by the small to large differentiated foliiform bracteoles at the base of the head. This contrasts with the loose but undiffentiated narrow involucral bracts at the base of the head in *Centrapalus* and the small and broad but otherwise undifferentated bracts at the base of the head in *Vernonella*.

On the basis of structural features, the genus is clearly distinct, but the position nearer *Centrapalus* of the subtribe Centrapalinae or closer to *Vernonella* of the Linziinae, has been resolved with certainty only with DNA sequencing. Sequence data are available for *Centrapalus*, *Parapolydora* and *Nothovernonia purpurea* (see below). Members of *Vernonella* have not been sequenced, but the subtribal placement is derived from the report of elemanolide sesquiterpene lactones from one of the species, *Vernonia praemorsa* (Muschl.) H.Rob. & Skvarla (Jakupovic et al. 1987), a type of sesquiterpene lactone known in the Vernonieae almost exclusively from the more basal members of the tribe.

## Phylogeny

Figure 2 is the result of the analysis of DNA sequence data and shows all of the subtribes in the Vernonieae and all the known genera within the Centrapalinae. Within this scheme, *Vernonia purpurea* belongs in the broad group first placed by Robinson (1999a) in the Erlangeinae, but subsequently shown by DNA sequencing to belong to the distinct subgroup seen in Keeley et al. (2007) containing *Centrapalus*, *Cabobanthus* H.Rob., *Parapolydora* H.Rob. (as *Vernonia fastigiata* Oliv. & Hiern in Oliv.), *Brachythrix* Wild & Pope, *Centauropsis* Bojer ex DC., *Hilliardiella* H.Rob., and *Polydora* Fenzl. This group is now recognized as part of the subtribe Centrapalinae H.Rob. (Keeley and Robinson 2009). Of the more restricted clade containing the closest relatives of *Centrapalus* and *Vernonia purpurea*,according to the DNA sequence results, *Cabobanthus* is the most basal in the group; it differs from the more highly nested genera by its lophate pollen. *Parapolydora*, the closest to *Centraplus*, shares the characters of numerous idioblasts on the achenes and the deeply divided or mostly solitary elongate cells of the setulae on the achenes. *Nothovernonia*, the genus named here based on *Vernonia purpurea*, falls somewhat outside the *Centrapalus/Parapolydora* group with strongly acuminate tips on the involucral bracts, foliiform bracts subtending the heads, and achenes with short narrow raphids, and more extensively fused elongate cells in the setulae. It is notable, however that the three closely related genera, *Nothovernonia*, *Centrapalus* and *Parapolydora* all share setulae with cells separated to at least one third of their length, more separated than in the less closely related genera of the Centrapalinae.

## Taxonomic Treatment

Genera known to be in the subtribe Centrapalinae based on DNA sequence data are shown in Figure 2.

### Key to the Genera known to be in the Centrapalinae

(for a key to the subtribes of Vernonieae see Keeley and Robinson 2009)

**Table:** 

1	Receptacles with paleae	*Centauriopsis*
–	Receptacles without paleae	2
2	Inner pappus reduced of 3–15 short easily caduous bristles	*Brachythrix*
–	Inner pappus of 30 or more well-developed rather persistent long capillary bristles	3
3	Inflorescence spiciform, with clusters of heads in axils of leaves	*Cabobanthus*
–	Inflorescence a spreading panicle	4
4	Heads 0.5 cm or less wide, with 12–30 florets; setulae of achene with paired cells united essentially to tip	5
–	Heads 0.5 cm or more wide, with 30 florets or more; setulae of achenes with paired cells distinctly separated at tips or from base	6
5	Plants mostly annuals; stems with simple or L-shaped hairs pappus bristles usually tawny, yellowish or green, rarely white; pollen triporate or with short colpi	*Polydora*
–	Perennials; stems, leaves, involucres and corolla lobes with large T-shaped hairs; heads with 12–20 florets; pappus white; pollen sublophate, tricolporate	*Hilliardiella*
6	Setulae of achenes with pairs of cells fused to a third or more of their length; heads with small or large foliose bracts at base; involucral bracts acuminate at tips	*Nothovernonia*
–	Setulae of achenes with cells separated or solitary for most of their length; heads without differentiated foliose bracts at base; involucral bracts mostly with narrowly attenuate tips	7
7	Base of stem decumbent; pappus bristles grayish or tawny; setulae of achenes with cells elongate but separated to near base	*Centrapalus*
–	Base of stem erect; pappus bristles whitish; setulae of achenes with only one of cells elongate and solitary from near base	*Parapolydora*

#### Nothovernonia

H.Rob & V.A.Funk gen. nov.

urn:lsid:ipni.org:names:77111570-1

http://species-id.net/wiki/Nothovernonia

##### Latin

*Ad Vernonella in habitis herbaceis et floribus purpureis simila sed capitulis base bracteoliferis in lobis corollarum distaliter spiculiferis et in grana pollinis sublophatis totaliter tricolporatis differt. A Centrapalus in basis erectis et bracteis involcri apiculatis lateraliter scariosis differt*.

##### Type.

*Vernonia purpurea* Sch.Bip. ex Walp.

##### Description.

Coarse branching herbs to 0.7 m tall, stems erect from base, distinctly ribbed, pilose with spreading simple hairs. Leaves alternate with petioles 0.2–2.0 mm long; leaf blades oblong to lanceolate, sparsely pilose above with prominent persistent bases on the hairs, lower surface coarsely and densely pilose on major veins, surface with many glandular dots, secondary veins pinnate, ca. 6 pairs. Inflorescences terminal and from axils of reduced upper leaves, distinctly cymiform with distinct short to long peduncles; heads broadly campanulate, with minute to large foliose bracts at base; involucral bracts in ca. 5 series, strongly gradate, appressed, ovate to narrowly oblong, with dark median stripe, apex apiculate with distinct dark or rarely pale awn, with pale scarious lateral margins, outer surface with numerous arachnoid hairs from median band, spreading tranversely as the head expands; receptacles flat, without pales or hairs. Florets ca. 30–65 per head; corollas purple, with lobes and upper throat exceeding the pappus at anthesis, glanduliferous on throat, lobes spiculiferous with stout straight hairs distally on outer surface; anther thecae with small sterile border at base, endothecial cells oblong with sinuous lateral walls, apical appendage triangular, firm. Style base with annuliform sclerified node, distally with stout spreading sweeping hairs covering backs of style branches and upper 1 mm of style shaft. Achenes prismatic, 8–10-ribbed, with numerous idioblasts on surface, with small narrow raphids, setulae with pairs of cells fused together beyond basal 1/3; pappus with inner series of many crowded capillary bristles, white or rufous, less than 2/3 as long as corollas; outer pappus series of shorter, crowded lanceolate scales.

Pollen grains ca. 40 mm in diam., tricolporate with colpi reaching poles, spinose, surface sublophate with perforated tectum continuous between colpi, bacculae single under each spine.

Chromosome number n = 9 (Jones 1979a, as *Vernonia purpurea* Sch.Bip. ex Walp.).

##### Etymology.

The new generic name, *Nothovernonia*, means “false *Vernonia*”.

#### Nothovernonia purpurea

(Sch.Bip. ex Walp.) H.Rob. & V.A.Funk comb. nov.

urn:lsid:ipni.org:names:77111572-1

http://species-id.net/wiki/Nothovernonia_purpurea

Vernonia purpurea  Sch.Bip. ex Walp., Rep. 2: 946 (1843). Type: Ethiopia, *Schimper 1197* (holotype P).
Vernonia inulifolia  Steud. ex Walp., Rep. 2:946 (1843). Type: Ethiopia, Sholoda, *Schimper 221* (holotype P, isotypes BM, K).
Vernonia jaceoides  A. Rich., Tent. Fl. Abyss. 1: 376 (1848). Type: Ethiopia, Chire, *Dillon s.n.* (holotype P).
Vernonia rigorata  S. Moore, J. Bot. 41: 155 (1903). Type: Kenya, Simba, *Kassner 724* (holotype BM).
Vernonia scabrida  C.H, Wright, Bull. Misc. Inf. Kew 1906: 21 (1906). Type: Malawi, Namasi, *Cameron 41* (holotype K).
Vernonia duemmeri  S. Moore, J. Bot. 52: 91 (1914). Type: Uganda, *Dummer 35* (syntype BM, isosyntype K), *Wilson 72* (syntype BM).
Vernonia pascuosa  S. Moore, J. Linn. Soc., Bot. 47: 263 (1925). Type: Angola, Uije, *Gossweiler 7404* (holotype BM).
Vernonia keniensis  R.E. Fr., Acta Hort. Berg. 9: 114 (1929). Type: Kenya, *Fries and Fries 948* (holotype UPS; isotype K).
Centrapalus purpureus  (Sch.Bip. ex Walp.) H. Rob., Proc. Biol. Soc. Wash. 112(1): 236 (1999).
Linzia purpurea  (Sch.Bip. ex Walp.) Isawumi, Comp. Newsl. 46: 40 (2008).

##### Distribution.

The species is known from the Sudan, Ethiopia, Kenya, Tanzania, and Uganda, south to Angola and Malawi and west to southern Senegal.

##### Specimens examined.

**Congo.** Bafuka (ueli), 1929, Steyaert77(US). **French Equatorial Africa.** Dans la Haute-Kotto (Oubangu-Chari-A.E.F.), 100 km NW Labuya, 1921–1923, herb. G. le Testu 4127(BM, US). **Malawi.** s.l.,1891, Buchanan 143 (K, US). Machinga District: Liwonde Forest Reserve, in *Brachystegia* woodland on steep hillside, 15°07'S, 35°23'E, 3000 ft., 11 Apr 1984, Christenson and Solubeni 1454(US). Ntcheu District: Golomati Road, 4 km E of road to Dedza, 14°50'S, 35°25'E, 20 Apr 1984, Christenson, Patel and Lipende 1477 (US). Lilongwe District: Dzalanyana Forest Reserve, about 8 km from entrance gate, 14°15'S, 33°25'E, 3000 ft, 2 May 1984, Christenson and Lipende 1493(US). **Senegal.** Tambacounda, Dindéfello, à proximité du campement touristique, dans le vallée de la cascade, 12°22'N, 12°19'W, 200 m, 22 Sep 1994, Sambou et Madsen, Goudiaby, Traoré and Laegaard 319 (AAU, DAKAR, US). **Uganda.** s.l., s.d.,Dummer 84(US, isotype of *Vernonia dummeri*). **Upper Volta.** Ifan, Solenso, 16 Sep 1974, Bognounou-Quattara 2(US). **Zambia.** Kitwe, 15 May 1967, DZF? F10,062(NDO, US).

##### Diagnostic characters.

Figure 3 illustrates the habit of *Nothovernonia purpurea*, note the large bracteoles at the base of the capitula. Figure 4 has the details of the capitula, note the stout hairs of the style branches (Fig. 4D) and the setulae of the achenes that have pairs of cells fused together beyond the basal 1/3 (Fig. 4H).

*Nothovernonia purpurea* has large, obvious foliiform bracteoles at the bases of the capitula, bracteoles that can often cover the involucre completely. In contrast, *Nothovernonia amblyolepis* has minute foliose bracts that are easily overlooked.

#### Nothovernonia amblyolepis

(Baker) H.Rob. & V.A.Funk comb. nov.

urn:lsid:ipni.org:names:77111573-1

http://species-id.net/wiki/Nothovernonia_amblyolepis

Vernonia amblyolepis  Baker, Bull. Misc. Inf. Kew 1898: 146 (1898). Type: Malawi, Nyika Plateau, *Whyte 204* (Lechtotype designated here, K, scanned image at US!), Mpata to Nyasa-Tanganyika Plateau, *Whyte s.n.* (isolectotype, K, scanned image at US!). The two specimens at K are labeled as syntypes. The Whyte 204 was selected as the lectotype because it is numbered.
Vernonia pratensis  Hiern, Cat. Afr. Pl. Welw. 1: 523 (1898) homonym illeg., non Klatt 1892 nec Drake 1897. Type: Angola, *Welwitsch 3364* (isotype K).
Vernonia kandtii  Muschl., Bot. Jahrb. Syst. 46: 87 (1911). Type: Rwanda, Niansa, *Kandt 69* (holotype B, destroyed, isotype EA).
Vernonia exasperata  H. Wild, Kirkia 11: 12 (1978), nom. nov. for *Vernonia pratensis* Hiern.

##### Distribution.

The species is known from Angola, Malawi, Rwanda and Tanzania.

##### Specimens Examined.

**Malawi.** Zomba District: Zomba Plateau, top of hillside above KuChawe Inn, on grassy slope, 15°20'S, 35°18'E, 5500 ft., 14 Apr 1984, Christenson 1461 (US). Zomba District: Zomba Plateau, on upper road from KuChawe Inn downward to Zomba, near horse paddock, 15°20'S, 35°18'E, 5000 ft., 25 Apr 1984, Christenson and Lipende 1488(US). **Uganda.** Buhweju County: District West Ankole, Nyarwambu River, 0°22'S, 30°28'E, 1550 m, 23 Aug 1982, Rwaburindora 919(MO, US).

##### Unrecognized taxa.

Jeffrey (1988) treated *Vernonia kandtii* as a separate species that included *Veronia pratensis* Hiern and *V. exasperata* H. Wild in its synonymy. *Vernonia kandtii* is separated by Jeffrey (1988), with some doubt, by his couplet 106 as follows:

**Table:** 

	Inflorescence copious, with a number of branches arising from the uppermost leaf-axils; ultimate peduncles short, so that the capitula appear to be in clusters	62. *Vernonia amblyolepis*
	Inflorescence strictly terminal, few-headed, lax, the ultimate peduncles longer, so that the capitula do not appear to be in clusters	61. *Vernonia kandtii*

The specimens from Malawi and Uganda that were examined for this study show variation in the structure of the inflorescence and therefore, do not support a separate species status for *Vernonia kandtii*.

## Supplementary Material

XML Treatment for Nothovernonia

XML Treatment for Nothovernonia purpurea

XML Treatment for Nothovernonia amblyolepis
